# Effects of capillary refill time-vs. lactate-targeted fluid resuscitation on regional, microcirculatory and hypoxia-related perfusion parameters in septic shock: a randomized controlled trial

**DOI:** 10.1186/s13613-020-00767-4

**Published:** 2020-11-02

**Authors:** Ricardo Castro, Eduardo Kattan, Giorgio Ferri, Ronald Pairumani, Emilio Daniel Valenzuela, Leyla Alegría, Vanessa Oviedo, Nicolás Pavez, Dagoberto Soto, Magdalena Vera, César Santis, Brusela Astudillo, María Alicia Cid, Sebastian Bravo, Gustavo Ospina-Tascón, Jan Bakker, Glenn Hernández

**Affiliations:** 1 Departamento de Medicina Intensiva, Facultad de Medicina, Pontificia Universidad Católica de Chile, Diagonal Paraguay 362, Santiago, Chile; 2 Unidad de Cuidados Intensivos, Hospital Barros Luco-Trudeau, Santiago, Chile; 3 Departamento de Medicina Interna, Facultad de Medicina, Universidad de Concepción, Concepción, Chile; 4 Department of Intensive Care Medicine, Fundación Valle del Lili, Universidad ICES, Cali, Colombia; 5 Division of Pulmonary, Allergy, and Critical Care Medicine, Columbia University Medical Center, New York, USA; 6 Department Intensive Care Adults, Erasmus MC University Medical Center, Rotterdam, CA The Netherlands; 7 Division of Pulmonary, and Critical Care Medicine, New York University-Langone, New York, USA

**Keywords:** Sepsis, Septic shock, Lactate, Hypoxia, Capillary refill time

## Abstract

**Background:**

Persistent hyperlactatemia has been considered as a signal of tissue hypoperfusion in septic shock patients, but multiple non-hypoperfusion-related pathogenic mechanisms could be involved. Therefore, pursuing lactate normalization may lead to the risk of fluid overload. Peripheral perfusion, assessed by the capillary refill time (CRT), could be an effective alternative resuscitation target as recently demonstrated by the ANDROMEDA-SHOCK trial. We designed the present randomized controlled trial to address the impact of a CRT-targeted (CRT-T) vs. a lactate-targeted (LAC-T) fluid resuscitation strategy on fluid balances within 24 h of septic shock diagnosis. In addition, we compared the effects of both strategies on organ dysfunction, regional and microcirculatory flow, and tissue hypoxia surrogates.

**Results:**

Forty-two fluid-responsive septic shock patients were randomized into CRT-T or LAC-T groups. Fluids were administered until target achievement during the 6 h intervention period, or until safety criteria were met. CRT-T was aimed at CRT normalization (≤ 3 s), whereas in LAC-T the goal was lactate normalization (≤ 2 mmol/L) or a 20% decrease every 2 h. Multimodal perfusion monitoring included sublingual microcirculatory assessment; plasma-disappearance rate of indocyanine green; muscle oxygen saturation; central venous-arterial pCO_2_ gradient/ arterial-venous O_2_ content difference ratio; and lactate/pyruvate ratio. There was no difference between CRT-T vs. LAC-T in 6 h-fluid boluses (875 [375–2625] vs. 1500 [1000–2000], *p* = 0.3), or balances (982[249–2833] vs. 15,800 [740–6587, *p* = 0.2]). CRT-T was associated with a higher achievement of the predefined perfusion target (62 vs. 24, *p* = 0.03). No significant differences in perfusion-related variables or hypoxia surrogates were observed.

**Conclusions:**

CRT-targeted fluid resuscitation was not superior to a lactate-targeted one on fluid administration or balances. However, it was associated with comparable effects on regional and microcirculatory flow parameters and hypoxia surrogates, and a faster achievement of the predefined resuscitation target. Our data suggest that stopping fluids in patients with CRT ≤ 3 s appears as safe in terms of tissue perfusion.

Clinical Trials: ClinicalTrials.gov Identifier: NCT03762005 (Retrospectively registered on December 3rd 2018)

## Background

Persistent hyperlactatemia has been traditionally considered as a signal of tissue hypoperfusion or hypoxia in septic shock patients [1]. Therefore, lactate normalization is recommended as a resuscitation target by recent guidelines [2]. However, hyperlactatemia is a non-specific marker of hypoperfusion, since other pathogenic mechanisms such as sustained hyperadrenergia and impaired hepatic clearance may contribute to increased serum lactate levels [1, 3, 4]. This may have relevant clinical implications, since if non-hypoperfusion-related sources predominate, increased serum lactate levels This may have relevant clinical implications, since if non-hypoperfusion-related sources predominate, pursuing lactate as a target may lead to fluid overload, potentially increasing mortality or morbidity [5–7]. In addition, kinetics of lactate recovery is relatively slow, which makes it a suboptimal target for fluid resuscitation [4, 8].

Peripheral perfusion appears as a promising alternative target [9, 10]. The excellent prognosis associated with capillary refill time (CRT) normalization [11], the rapid-response time to fluid loading [8], plus its simplicity and availability in resource-limited settings, constitute a solid background to promote studies evaluating its usefulness to guide fluid resuscitation. The ANDROMEDA-SHOCK trial was implemented within 4 h of septic shock diagnosis and compared a CRT-vs. a lactate-targeted resuscitation strategy [12–14]. The CRT group exhibited a non-significant lower mortality (34.9 vs. 43.4%, *p* = 0.06), required less fluid resuscitation during the intervention period, and presented less organ dysfunction at 72 h. A posterior Bayesian analysis showed a very high probability that CRT-targeted resuscitation may result in lower mortality and faster resolution of organ dysfunction compared to a lactate-targeted one [15]. ANDROMEDA-SHOCK results should be confirmed by future major trials, but in the meantime, many non-resolved issues could be addressed by smaller randomized controlled trials including the effect of both strategies on organ perfusion.

We designed the present trial to address the impact of a CRT-targeted vs. lactate-targeted fluid resuscitation strategy started within 24 h after septic shock diagnosis on fluid administration and balances. In addition, we aimed at comparing the effects of both strategies on organ dysfunction, regional and microcirculatory flow, and tissue hypoxia surrogates.

## Materials and methods

### Study design

This was a prospective randomized controlled trial conducted at the intensive care units (ICU) of two teaching hospitals, Hospital Clínico UC CHRISTUS and Hospital Barros Luco-Trudeau of Santiago, Chile. The study was approved by the Institutional Review Board of both centers. A signed informed consent was asked to the next of kin of all eligible patients and confirmed by the patients when feasible.

### Patient selection and randomization

Consecutive adult patients (≥ 18 years) with septic shock as defined by a serum lactate > 2 mmol/liter and requirements of norepinephrine (NE) to maintain a mean arterial pressure (MAP) ≥ 65 mmHg after an intravenous fluid load of at least 20 ml/kg over 60 min [16], and with a demonstrated fluid-responsiveness state [17] were considered as eligible. Patients had to be recruited within a period of 24 h after septic shock diagnosis. Exclusion criteria were pregnancy, anticipated surgery or dialytic procedure during the first 6 h after potential inclusion, active bleeding, Child B or C liver cirrhosis, severe acute respiratory distress syndrome, and do-not-resuscitate status.

Eligible patients were randomly allocated to CRT-targeted (CRT-T) or lactate-targeted (LAC-T) fluid resuscitation arms. A randomization sequence by permuted blocks of eight with an allocation of 1:1 was generated by a computer program. Allocation concealment was maintained by means of central randomization.

### Study interventions

The intervention period was of 6 h. Before starting the study, all centers were trained to assess CRT with a standardized technique. Briefly, CRT was measured by applying firm pressure to the ventral surface of the right index finger distal phalanx with a glass microscope slide. The pressure was increased until the skin was blank and then maintained for 10 s. The time for return of pre-existent skin color was registered with a chronometer, and a CRT higher than 3 s was defined as abnormal [13].

The perfusion target for CRT-T was a normal CRT (≤ 3 s). The perfusion target for LAC-T was an arterial lactate ≤ 2 mmol/l or a decrease > 20% every 2 h. CRT was assessed every 30 min and lactate every 2 h during the intervention period, after which the treatment was liberalized for attending physicians.

Fluid responsiveness was assessed with different techniques according to usual practice and clinical context. Cut-offs to consider a patient as fluid responsive for each technique are shown in Additional file 1 [18].

The single intervention of the study was the administration of repeated fluid boluses. The single intervention of the study was the administration of repeated fluid boluses. Fluids (500 ml of Ringer's lactate administered in 30-min intervals) were repeated until the perfusion target was achieved, or fluid responsiveness became negative, or a safety limit of an increase in central venous pressure (CVP) ≥ 5 mmHg after a fluid bolus was reached [19].

When the perfusion target could not be achieved with fluids, other resuscitation steps such as addition or modulation of vasoactive agents, or potential rescue therapies were decided by attending physicians. Besides sepsis source aggressive management, all patients were treated as recommended by current guidelines [2].

### Measurements and data collection

Clinical and demographic variables were registered at baseline (T0). All patients were followed until hospital discharge. All data including demographic aspects, sepsis sources and management, inflammatory biomarkers, severity scores and major outcomes were registered.

For this research protocol, several specific research-related variables were measured or calculated at baseline, 2 (T2), 6 (T6) and at 24 h (T24).

Hemodynamic and clinical perfusion variables included fHemodynamic and clinical perfusion variables included fluid boluses together with total fluid inputs/outputs and fluid balances; macrocirculatory variables such as MAP, heart rate, CVP, NE dose, macrocirculatory, cardiac output (CO) assessed with non-invasive pulse-contour technique (PiCCO device, Pulsion Medical Systems, Munich, Germany) or a pulmonary artery catheter; c, Pulsion Medical Systems, Munich, Germany; perfusion variables such as arterial lactate, central venous oxygen saturation (ScvO_2_), and central venous-arterial pCO_2_ gradient (P(cv-a) CO_2_); and; and CRT. CRT.

In addition, regional and microcirculatory perfusion-related variables were assessed. In addition, regional and microcirculatory perfusion-related variables were assessed. Sublingual microcirculation was evaluated with the side dark field (SDF) device. At each assessment, at least five 10-20 s video images were recorded. The analysis was performed by eye by an expert researcher following recent recommendations [20]. From image analyses, the microcirculatory flow index (MFI) was calculated. A MFI ≤ 2.5 was considered as abnormal following some previous reports [3, 21]. Plasma-disappearance rate of indocyanine green (PDR-ICG) was determined with a non-invasive transcutaneous assessment of ICG clearance to indirectly assess liver blood flow [22]. An ICG finger clip was fixed in every patient and then connected to a liver function monitor (LiMON; Pulsion Medical Systems, Munich, Germany). A dose of 0.25 mg/kg of ICG was then injected through a central venous catheter. Normal range for PDR-ICG is 18% to 25% per min with a value < 15%/min considered as abnormal in some previous studies [22, 23]. 

Near infrared spectroscopy (NIRS): Muscle oxygen saturation (StO_2)_ was assessed by a tissue spectrometer (InSpectra Model 325; Hutchinson Tc, Mn, USA). A NIRS probe was placed on the skin of the thenar eminence. StO_2_ < 70% is abnormal [24]. SDF and PDR-ICG were only performed at the Hospital Clínico UC CHRISTUS SDF and PDR-ICG were only performed at the Hospital Clínico UC CHRISTUS.

Two hypoxia-related indexes were also assessed: (1) Central venous-arterial pCO_2_ gradient/arterial-venous O_2_ content difference ratio (P(cv-a)CO_2_/Da-vO_2_): This ratio was calculated after taking arterial and central venous blood gases. A ratio ≥ 1.4 may be associated to anaerobic CO_2_ generation [25–27]. (2) Lactate/pyruvate (L/P) ratio: Arterial blood samples for pyruvate were taken with immediate deproteinization of the sample, and processed in our laboratory before 3 h by enzymatic fluorometric-assay (Sigma-Aldrich, USA). A L/P ratio > 15 suggests tissue hypoxia in some previous work [28]. in some previous work Both ratios may represent an expression of anaerobic metabolism at the cellular level and thus, may be used as hypoxia surrogates.

## Outcome measures 

The primary outcome was fluid volume administered during the 6 h intervention period. Secondary prespecified outcomes included fluid balance at 24 h; 24 h SOFA score; and previously mentioned regional, microcirculatory flow, and tissue hypoxia surrogates.

## Sample size calculation

After a thorough literature review, we found only one pilot study comparing peripheral perfusion vs. standard care based resuscitation in septic shock patients [10], showing that the former resulted in significantly less resuscitation fluids at 6 h (4227 ± 1081 ml vs. 6069 ± 1715 ml). In consequence, we considered a 1600 ml difference in primary outcome between study groups to be the critical threshold for hypothesis testing. If there was no difference between standard and experimental treatments, then 46 patients would be required (23 patients per arm) to be 90% sure that the lower limit of a two-sided confidence interval was above the limit of − 1600 mL at an alpha level of 0.05.

## Statistical analysis

As variables presented non-normal distribution, non-parametric tests were used. Descriptive statistics are presented as median [interquartile range] or percentage. Mann–Whitney U Test, chi-square or Fisher’s exact test were used when appropriate. Two-tailed p value < 0.05 was considered significant. Data were analyzed with Minitab v17 (Minitab Inc, State College, PA) and Graphpad Prism (Graphpad Softwares, La Joya, CA) softwares.

## Results

### Patients characteristics

**Fig. 1:**
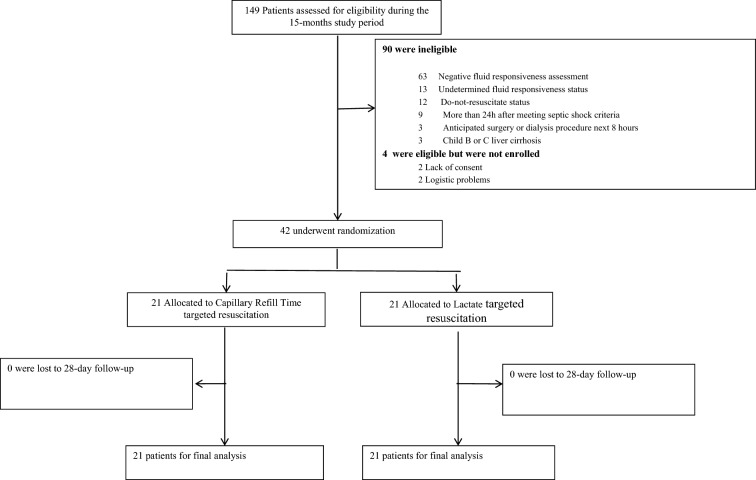
Study flow diagram

This study was conducted between June 2018 and October 2019, where it was stopped before completing the programmed sample size of 46 patients. The decision was made because of nil further recruitment after the start of a severe Chilean social outburst in October. During the study period, 149 patients were screened for potential protocol inclusion. Patient flow and causes for exclusion are represented in Fig. 1. Finally, forty-two patients were randomized to both study arms.

Baseline demographic, severity scoring, hemodynamic and perfusion characteristics are shown in Table 1. Time from septic shock diagnosis to protocol start was similar in both arms (CRT-T, 4 [2–9] h vs. LAC-T, 5 [2–6] h; *p* = 0.9). The most common tests for fluid responsiveness assessment were pulse pressure variation (CRT-T, 43% vs. LAC-T, 52%; *p* = 0.8), inferior vena cava variation (CRT-T, 29% vs. LAC-T, 19%; *p* = 0.4), and passive leg raising with velocity–time integral (CRT-T, 10% vs. LAC-T, 19%; *p* = 0.6).

**Table 1:** Baseline characteristics of the study population

	CRT targeted group	Lactate targeted group
N°	21	21
Age (years)	51 [45–75]	66 [55–75]
Female	48 (10)	66 (14)
APACHE score	23 [15–30]	23 [14–34]
SOFA score	11 [8–14]	12 [9–14]
Septic Source	Abdominal: 62 (13)	Abdominal 52: (11)
	Other: 14 (3)	Other: 10 (2)
	Respiratory: 14 (3)	Respiratory: 14 (3)
	Urinary: 10 (2)	Urinary: 24 (5)
Surgical Source	57 (12)	62 (13)
Time to antibiotics (min)	60 [45–120]	60 [30–60]
Fluids Pre-randomization (ml)	1550[1000–3000]	2500 [1250–3175]
MV at inclusion	95 (21)	81 (17)
MAP (mmHg)	71 [66–77]	69 [61–78]
CVP (mmHg)	8 [5–12]	8 [6–10]
Cardiac Index (l/m/m^2^)	3.1 [2.2–3.5]	2.8 [1.9–3.9]
Norepinephrine dose (mcg/kg/min)	0.23 [0.14–0.54]	0.29 [0.16–0.4]
Lactate (mmol/L)	3 [2.8–5.5]	4 [3–7.6]
CRT (s)	5 [3–6.5]	5 [3–6.5]
ScvO_2_ (%)	69 [65–78]	70 [59–79]
Delta pCO_2_(v-a)	8 [4–13]	7 [5–11]
P(cv-a)CO_2_/Da-vO_2_ ratio	2 [1–2.5]	1.3 [1.1–2.8]
L/P ratio	8.1 [4–13.9]	9 [4.7–14]
StO_2_ (%)	78 [66–82]	78 [70–83]
PDR-ICG^a^ (%)	18 [10–21.8]	12 [10–15.4]
MFI^a^	1.75 [1–2.5]	2.6 [2.5–3]

Data are presented as percentage (absolute number) or median [interquartile range]

*CRT* Capillary refill time, *APACHE* *II* Acute Physiology And Chronic Health Evaluation II, *SOFA* Sequential organ failure Assessment score, *MV* Mechanical ventilation, *MAP* Mean arterial pressure, *CVP* Central venous pressure, *ScvO*_*2*_ central venous oxygen saturation, *Delta pCO*_*2*_*(v-a)* Difference between central venous carbon dioxide pressure and arterial carbon dioxide pressure, *P(cv-a)CO*_*2*_*/Da-vO*_*2*_ ratio central venous-arterial pCO_2_ gradient/ arterial-venous, *O*_*2*_ content difference ratio, *L/P ratio *lactate-piruvate ratio, *StO*_*2*_ Thenar muscle oxygen saturation, *PDR-ICG* Indocianine greeen plasma disappearance rate,*MFI* Microcirculatory flow index

^a^Assessed only at Hospital Clínico UC CHRISTUS

## Study outcomes

**Fig. 2:**
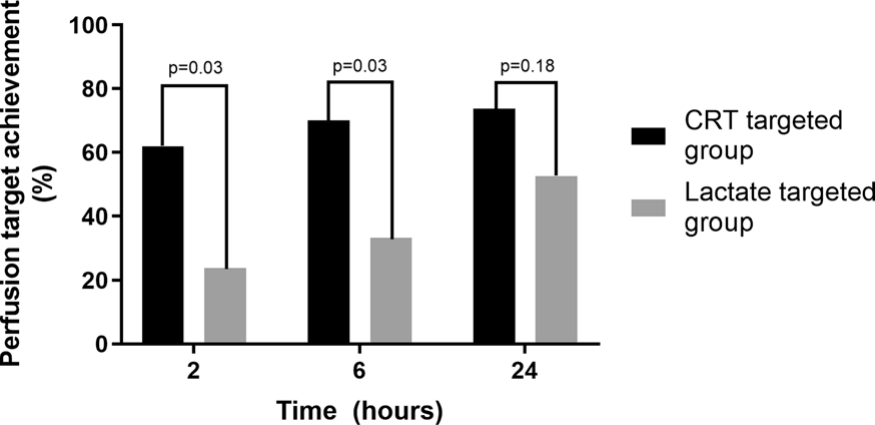
Achievement of perfusion target according to study arm at 2, 6 and 24 h. CRT: Capillary refill time

Table 2 shows a comparison between both study groups according to 6 h fluid boluses and balances, 24 h SOFA, and perfusion targets. No significant difference was observed between groups in the primary outcome of resuscitation fluid administration at 6 h. In addition, there was no difference between CRT-T and LAC-T groups in 24 h SOFA score (10 [5–13] vs. 11 [7–13], *p* = 0.8), 28- day mortality (24% vs. 19%, *p* = 0.8), ICU (6 [5–14] vs. 10 [4–20] days, *p* = 0.8) and hospital length of stay (17[6–58] vs. 26 [9–53] days, *p* = 0.9). Concerning specific resuscitation targets at 6 h, a higher proportion of patients in the CRT-T group achieved their objective (62 vs. 24, *p* = 0.03), as shown in Fig. 2.

**Table 2:** Fluid administration, balance, perfusion targets and vasopressor dosage in both randomization arms during the study period

	CRT targeted group	Lactate targeted group	p-value
Fluid Bolus 6 h (ml)	875 [375–2625]	1500 [1000–2000]	0.3
Fluid Balance 6 h (ml)	982 [249–2833]	1580 [740–6587]	0.2
Fluid Balance 24 h (ml)	1710 [614–4172]	2015 [166–5060]	0.85
CRT 6 h (s)	3 [2–5]	3 [2–5]	0.8
CRT 24 h (s)	3 [2–4]	3 [2, 3]	0.9
Lactate 6 h (mmol/L)	2.3 [1.8–4]	3.5 [1.8–7.3]	0.4
Lactate 24 h (mmol/L)	2 [1.3–3.2]	1.9 [1.4—4.3]	0.9
NE dose 6 h (mcg/kg/min	0.33 [0.11–0.46]	0.21 [0.12–0-35]	0.26
NE dose 24 h (mcg/kg/min)	0.14 [0.04–0.32	0.13 [0.01–0.25]	0.6

Data are presented as median [interquartile range]

*CRT* Capillary refill time, *NE* norepinephrine

## Multimodal perfusion assessment

When assessing regional, microcirculatory and hypoxia-related parameters, no difference between groups was observed at the end of the intervention period, as shown in Table 3. Additional file 2 shows the number of tests performed at each time point in both study groups.

**Table 3:** Multimodal perfusion comparison between both study groups at 6 h

	CRT-targeted group	Lactate targeted group	P
MAP (mmHg)	70 [65–65]	73 [64–81]	0.6
CVP (mmHg)	9 [7–12]	10 [8–13]	0.54
Cardiac Index (l/m/m^2^)	2.7 [2.2–4]	3.1 [2.5–3.9]	0.4
Norepinephrine dose (mcg/kg/min)	0.33 [0.11–0.46]	0.21 [0.12–0.35]	0.26
Lactate (mmol/L)	2.3 [1.8–4]	3.5 [1.8–7.3]	0.37
CRT (s)	3 [2–5]	3 [2–5]	0.82
ScvO_2_ (%)	68 [66–76]	73 [61–82]	0.38
Delta pCO_2_(v-a)	5 [4–8]	5 [4–8]	0.89
P(cv-a)CO_2_/Da-vO_2_ ratio	1.58 [1.1–2]	1.8 [1–2.7]	0.6
L/P ratio	12 [6.1–22.2]	8.7 [4.5–12.4]	0.22
StO_2_ (%)	75 [70–85]	80 [68–85]	0.6
PDR-ICG^a^ (%)	15.6 [12–24]	13 [6–14]	0.1
MFI^a^	2.5 [1.5–3]	3 [2.7–3]	0.09

Data are presented as percentage (absolute number) or median [interquartile range]

*CRT* Capillary refill time, *MAP* mean arterial pressure, *CVP* Central venous pressure, *ScvO*_*2*_ central venous oxygen saturation, *Delta pCO2(v-a)* Difference between central venous carbon dioxide pressure and arterial carbon dioxide pressure, *P(cv-a)CO2/Da-vO2 *ratio central venous-arterial pCO_2_ gradient/ arterial-venous O_2_ content difference ratio, *L/P ratio *lactate-piruvate ratio, *StO*_*2*_ Thenar muscle saturation, *PDR-ICG* Indocianine greeen plasma disappearance rate, *MFI *microcirculatory flow index

^a^Assessed only at Hospital Clínico UC CHRISTUS

## Discussion

CRT-targeted fluid resuscitation was not superior to a lactate-targeted one in the primary outcome of fluid administration during the 6 h intervention period, neither in fluid balances nor organ dysfunction at 24 h. However, CRT-targeted resuscitation was associated with higher achievement of resuscitation targets during the intervention period and exhibited comparable effects to LAC-T on regional/microcirculatory flow parameters and hypoxia-surrogates.

Our results could be viewed as in contradiction with the findings of the ANDROMEDA-SHOCK trial [14], particularly in the fluid boluses administered during the intervention period. However, the design of the studies was markedly different. First, the time-period for recruitment was maximum 4 h, since septic shock diagnosis in ANDROMEDA-SHOCK and up to 24 h in the present study. Fluid boluses administered in CRT-T were almost a half of those in LAC-T, and although this difference is not significant, it may suggest that the benefits of CRT-guided resuscitation may extend for longer periods than the limits imposed by ANDROMEDA-SHOCK. Second, in the present study only fluid-responsive patients were included, and in addition it included fluid administration as the single intervention. Despite these differences in design, LAC-T, the challenged gold-standard, was not superior in any of the studied variables, and patients in CRT-T achieved their goal in a higher proportion of cases during the intervention period.

A possible explanation of our findings is that since CRT exhibits a rapid response to flow increasing maneuvers it could be assessed in periods of 30 min thus allowing clinicians to stop resuscitation in a timely fashion. In contrast, lactate exhibits a relatively slow and biphasic recovery kinetics even after successful resuscitation [3], thus being associated with the potential risk of fluid overload. This fact was behind the working hypothesis of the previous ANDROMEDA-SHOCK study [14].

Concerning the impact of both strategies on regional and microcirculatory flow, to the best of our knowledge this is the first study assessing this issue with a multimodal approach. In a previous study, Brunauer et al. found a significant correlation between changes in CRT with the pulsatility index of various hepatosplanchnic arteries during septic shock resuscitation [29]. This is physiologically coherent since both territories are affected by the adrenergic response to shock that could be reverted at least partially by increments in systemic flow. Additionally, another study compared dobutamine vs. placebo on regional and microcirculatory flow in hyperdynamic septic shock, demonstrating that patients who normalized CRT exhibited also normal muscle O_2_ saturation and plasma disappearance rate for indocyanine green [21]. Although not powered for secondary outcomes, our study suggests that CRT-targeted resuscitation at least does not deteriorate liver blood flow, muscle oxygen saturation, and sublingual microvascular flow in comparison to a lactate-targeted one. In fact, it appears that both strategies lead to a similar improvement in these variables which adds new information concerning the safety of targeting peripheral perfusion during septic shock resuscitation.

Another peripheral perfusion assessment method is the mottling score [30]. Its prognostic value, pathophysiologic correlates [31, 32], as well as its relationship with tissue perfusion has been clearly demonstrated [29]. Mottling score may be complementary to CRT for a thorough assessment of peripheral perfusion, but less data are available on its kinetics of recovery after fluid resuscitation. This is the main reason why CRT was selected as a target in ANDROMEDA-SHOCK and the present study.

None of the classic perfusion-related parameters reliably reflect the presence or absence of tissue hypoxia. Tissue hypoxia should englobe the idea of an impaired critical oxygen delivery, and/or the inability of the mitochondria to utilize O_2_, leading to an exclusive anaerobic metabolism in affected territories [33]. Consequently, both O_2_ consumption and aerobic CO_2_ production are decreased and a critical amount of anaerobic CO_2_ is generated due to massive ATP degradation, with the resulting buffering of free H^+^ with plasma HCO_3_^−^. Once tissue hypoxia is established, the reduction of cell redox potential shifts the production of ATP to the anaerobic pathways, elevating the L/P ratio [33] and furthermore increasing anaerobic CO_2_ production. This shift could rise the respiratory quotient and consequently the P(cv-a)CO_2_/ Da-vO_2_ ratio [26]. Both indexes could theoretically be used as surrogates of tissue hypoxia and some previous studies showed their relationship with hyperlactatemia and progressive shock [34], although the subject is controversial [26]. In our study, we observed no differences between groups on these hypoxia surrogates, again suggesting that targeting normal CRT appears as safe at the tissue level as compared with the gold standard lactate-targeted resuscitation.

Our study has several limitations. First, there are inherent technical drawbacks and interpretation issues for each of the flow-related variables assessed in the study. PDR-ICG depends basically on liver blood flow but also on liver metabolic function [22]. Thus, pre-existing or acute liver dysfunction precludes a correct interpretation of results. We excluded patients with advanced liver dysfunction but cannot rule-out some degree of subclinical dysfunction. In addition, the ICG finger clip may loose the signal in the presence of profound peripheral vasoconstriction. Muscle StO_2_ is flow-sensitive and was described in hemorrhagic shock, where it improves after successful resuscitation [35]. StO_2_ decreases rapidly after a vascular occlusion test and recovers very fast after releasing compression [21, 24]. However, its role in hyperdynamic states is uncertain. On the other hand, after almost two decades of initial description, sublingual microcirculatory assessment has not been moved to routine clinical practice and is still positioned in the research arena [38]. Technical aspects, logistics, costs, and lack of agreement between experts on the best way to take and analyze images have precluded further development [20]. In this study, we simplified analyses considering only MFI which is a flow-related parameter and the easiest to standardize.

Second, in the case of hypoxia surrogates, the background literature is scarce and both ratios are not universally accepted. Additional problems are the technical difficulties for assessing pyruvate, and the lack of clear cut-offs for abnormality. In the case of P(cv-a)CO_2_/ Da-vO_2_, the use of central venous instead of mixed venous pCO_2_, and the use of differences in pressures and not CO_2_ contents could be criticized [26]. However, the decision to use the simplified ratio was for practical reasons and has supportive literature [25, 26].

Third, the small sample size may be a problem in physiologically focused studies. Therefore, we consider these results only as hypothesis-generating, but the data obtained may aid in bringing insights into the mechanisms behind the positive prognostic value of a normal CRT after initial fluid resuscitation. Fourth, CRT assessment might be subjected to inter-observer variability, but we used a standardized technique that decreases the likelihood of bias. Fifth, the premature stop of the study may have introduced bias but an independent statistician analyzed the results and concluded that recruitment of 4 more patients would not have changed the p values of the main findings. Finally, we could perform SDF and PDR-ICG techniques in only one of the two involved centers for logistic reasons, but we think that this does not invalidate our conclusions.

## Conclusions

CRT-targeted fluid resuscitation in septic shock was not superior to a lactate-targeted one on early fluid administration or fluid balances. However, it was associated with comparable effects on regional and microcirculatory flow parameters and hypoxia surrogates. In addition, achievement of allocated targets was higher for CRT-guided resuscitation during the 6 h intervention period. These data, although only hypothesis generating, expand the results of ANDROMEDA-SHOCK suggesting that potential benefits of CRT-targeted resuscitation should be tested in future studies beyond the limits of very early septic shock.

## Supplementary information

**Additional file 1: **Methods used in the and cut-offs for fluid responsiveness assessment techniques in the present study

**Additional file 2:** Proportion of target achievers at 6h with normal values of perfusion-related variables in both study arms

## Data Availability

The datasets generated and/or analyzed during the current study are not publicly available until August 2020, but are available before from the corresponding author on reasonable request.

## Abbreviations

CRT: Capillary refill time
CRT-T: Capillary refill time targeted fluid resuscitation
LAC-T: Lactate-targeted fluid resuscitation
ICU: Intensive care unit
MAP: Mean arterial pressure
NE: Norepinephrine
LOS: Length of stay;
APACHE: Acute Physiology and Chronic Health Evaluation
SOFA: Sequential Organ Failure Assessment
ScvO_2_: Central venous oxygen saturation
Delta pCO2(v-a): Difference between central venous carbon dioxide pressure and arterial carbon dioxide pressure
P(cv-a)CO2/Da-vO2 ratio: Central venous-arterial pCO_2_ gradient/ arterial-venous O_2_ content difference ratio
L/P ratio: Lactate/piruvate ratio
StO_2_: Thenar muscle saturation
PDR-ICG: Plasma disappearance rate of indocyanine green
MFI: Microcirculatory flow index
