# Supplementary material for: Effects of capillary refill time-vs. lactate-targeted fluid resuscitation on regional, microcirculatory and hypoxia-related perfusion parameters in septic shock: a randomized controlled trial
Source: Ann Intensive Care. 2020 Nov 2;10:150. doi: 10.1186/s13613-020-00767-4 (PMC7606372; doi:10.1186/s13613-020-00767-4)
Supplement: Supplementary file 1 — Additional file 1: Methods used in the and cut-offs for fluid responsiveness assessment techniques in the present study [file 13613_2020_767_MOESM1_ESM.docx]

Additional File 1.- Cutoff values of different fluid responsiveness assessment techniques

| Test | Details |
| --- | --- |
| PPV | Measured with the formula: (PPmax-PPmin)/((PPmax+PPmin)/2). Value > 13% was considered significant. |
| PLR VTI | Measured with the formula: (VTImax-VTImin)/ (VTImax+VTImin)/2). Value > 15% considered significant. |
| IVCV | With M-Mode, maximal and minimal diameter of IVC were registered. Value are obtained with the formula (Dmax-Dmin)/Dmin). Values > 15% are considered significant during mechanical ventilation . |

PPV: Pulse pressure variation; PLR-VTI: Passive leg raising with velocity time integral; IVCV: Inferior vena cava variation.
