# Supplementary material for: Effects of capillary refill time-vs. lactate-targeted fluid resuscitation on regional, microcirculatory and hypoxia-related perfusion parameters in septic shock: a randomized controlled trial
Source: Ann Intensive Care. 2020 Nov 2;10:150. doi: 10.1186/s13613-020-00767-4 (PMC7606372; doi:10.1186/s13613-020-00767-4)
Supplement: Supplementary file 2 — Additional file 2: Proportion of target achievers at 6h with normal values of perfusion-related variables in both study arms [file 13613_2020_767_MOESM2_ESM.docx]

**Additional File 2: Number of tests performed of each variable at different study timepoints**

|  | CRT-targeted group | | | Lactate targeted group | | |
| --- | --- | --- | --- | --- | --- | --- |
|  | 0 | 6 | 24 | 0 | 6 | 24 |
| Lactate | 21/21 | 21/21 | 20/20 | 21/21 | 21/21 | 19/19 |
| CRT | 21/21 | 21/21 | 20/20 | 21/21 | 21/21 | 19/19 |
| ScvO_2_ | 21/21 | 21/21 | 20/20 | 21/21 | 21/21 | 19/19 |
| Delta pCO_2_(v-a) | 21/21 | 21/21 | 20/20 | 21/21 | 21/21 | 19/19 |
| P(cv-a)CO_2_/Da-vO_2_ ratio | 16/21 | 16/21 | 15/20 | 15/21 | 15/21 | 13/19 |
| L/P ratio | 17/21 | 14/21 | 14/20 | 18/21 | 17/21 | 15/19 |
| StO_2_ | 21/21 | 21/21 | 21/21 | 20/21 | 20/21 | 19/19 |
| PDR-ICG* | 10/10 | 10/10 | 10/10 | 10/10 | 10/10 | 8/9 |
| MFI* | 9/10 | 9/10 | 9/10 | 8/10 | 8/10 | 8/9 |

* Assessed only at Hospital Clínico UC CHRISTUS

CRT: Capillary refill time; ScvO_2_: central venous oxygen saturation; Delta pCO2(v-a): difference between central venous carbon dioxide pressure and arterial carbon dioxide pressure; P(cv-a)CO2/Da-vO2 ratio: central venous-arterial pCO_2_ gradient/ arterial-venous O_2_ content difference ratio; L/P ratio:lactate-piruvate ratio; StO_2_: thenar muscle saturation; PDR-ICG: Indocianine greeen plasma disappearance rate; MFI: microcirculatory flow index.
